# Genome expansion of an obligate parthenogenesis-associated *Wolbachia* poses an exception to the symbiont reduction model

**DOI:** 10.1186/s12864-019-5492-9

**Published:** 2019-02-06

**Authors:** A. A. Kampfraath, L. Klasson, S. Y. Anvar, R. H. A. M. Vossen, D. Roelofs, K. Kraaijeveld, J. Ellers

**Affiliations:** 1 Department of Ecological Science, Vrije Universiteit Amsterdam, Amsterdam, The Netherlands; 2 Department of Cell and Molecular Biology, Uppsala University, Uppsala, Sweden; 3 Department of Human Genetics, Leiden University Medical Center, Leiden, The Netherlands; 4 Leiden Genome Technology Center, Leiden University Medical Center, Leiden, The Netherlands

**Keywords:** *Folsomia candida*, *Leptopilina clavipes*, *w*Fol, *w*Lcla, Parthenogenesis induction

## Abstract

**Background:**

Theory predicts that dependency within host-endosymbiont interactions results in endosymbiont genome size reduction. Unexpectedly, the largest *Wolbachia* genome was found in the obligate, parthenogenesis-associated *w*Fol. In this study, we investigate possible processes underlying this genome expansion by comparing a re-annotated *w*Fol genome to other *Wolbachia* genomes. In addition, we also search for candidate genes related to parthenogenesis induction (PI).

**Results:**

Within *w*Fol, we found five phage WO regions representing 25.4% of the complete genome, few pseudogenized genes, and an expansion of DNA-repair genes in comparison to other *Wolbachia*. These signs of genome conservation were mirrored in the *w*Fol host, the springtail *F. candida*, which also had an expanded DNA-repair gene family and many horizontally transferred genes. Across all *Wolbachia* genomes, there was a strong correlation between gene numbers of *Wolbachia* strains and their hosts. In order to identify genes with a potential link to PI, we assembled the genome of an additional PI strain, *w*Lcla. Comparisons between four PI *Wolbachia,* including *w*Fol and *w*Lcla, and fourteen non-PI *Wolbachia* yielded a small set of potential candidate genes for further investigation.

**Conclusions:**

The strong similarities in genome content of *w*Fol and its host, as well as the correlation between host and *Wolbachia* gene numbers suggest that there may be some form of convergent evolution between endosymbiont and host genomes. If such convergent evolution would be strong enough to overcome the evolutionary forces causing genome reduction, it would enable expanded genomes within long-term obligate endosymbionts.

**Electronic supplementary material:**

The online version of this article (10.1186/s12864-019-5492-9) contains supplementary material, which is available to authorized users.

## Background

Endosymbiotic bacteria are found in the cells of many eukaryotic species, where they affect major processes such as host reproduction [1, 2], defence against pathogens [3] and development [4, 5]. Endosymbionts are dependent on their host for proliferation, whereas host are not necessarily dependent on their endosymbiont and can often reproduce and survive in their absence. However, there are ample examples of obligate endosymbionts, e.g. *Buchnera* in aphids [6, 7], *Wigglesworthia* in the tsetseflies [8], and *Wolbachia* in parasitic filarial nematodes [9, 10]. In these cases neither host nor endosymbiont are viable without the other, and these associations are usually characterised by a long evolutionary history, nutritional or developmental dependency, and vertical transmission of the symbiont [11].

Current theory predicts that mutual dependency between host and endosymbiont leads to a reduction in genome size of endosymbionts [12, 13]. Different genetic mechanisms have been implied as the driving forces behind these reductions. First, co-adaptation between the symbiont and their host may result in redundancy of certain symbiont functions releasing genes from selective constraints, causing them to decay and eventually to disappear [14, 15]. Second, small population sizes and limited opportunity for horizontal gene exchange increases genetic drift to a level that purifying selection cannot overcome, which leads to deleterious mutations that cause pseudogenization of mildly advantageous genes and the eventual removal of those genes due to the inherent deletion bias found for bacterial genomes [12, 16, 17]. In addition, multiple transitional events seem to pinpoint moments of sever genome reduction [18]. These include becoming host bound, moving into a specialized host cell and being vertically transmitted through host generations. Intervals between different transitional events seem prone to different sets of selection pressures that influence genome size. For example, the negative correlation between host dependence and symbiont genome size only holds for vertically transmitted endosymbionts [19]. However, there is still a large amount of variation in the genome size of vertically transmitted endosymbionts that are not residing in specialized host cells, which is difficult to explain in the context of current theory [18]. Examining endosymbiont genomes in this stage will be needed to better understand all factors that influence the genome reduction during the evolution of endosymbionts.

*Wolbachia* is one of the most widespread endosymbionts [20, 21] and known for its variety of interactions with its host, including male killing, feminization, cytoplasmic incompatibility (CI), parthenogenesis-induction (PI) and provisional mutualisms [22, 23]. Plus, all known *Wolbachia* strains are in a similar transitional stage according to the classification of Lo et al. (2016), i.e. they are mainly transmitted vertically and do not reside in specialized structures. They are thus predicted to vary in genome size depending on the host dependency [18]. This prediction seems to hold for most of the *Wolbachia* strains, with reduced genomes that lack mobile elements found in obligate mutualistic strains and larger genomes in facultative reproduction-manipulating strains. However, recently a *Wolbachia* genome was sequenced that poses an exception to this pattern. The *Wolbachia* from the parthenogenetic collembolan host *Folsomia candida* has the largest *Wolbachia* genome sequenced so far [24], yet it is obligate to its host. Since, elimination of *Wolbachia* by heat or antibiotics, renders the host’s eggs non-viable [25, 26]. In addition, the *w*Fol genome is the first fully sequenced genome from supergroup E that is positioned to be a sister group to supergroup A, B, C, D, F and H, and shares a more ancestral common ancestor with supergroup L [27, 28]. All complete genomes from members of these supergroups have smaller genomes than *w*Fol, which suggests that this obligate *Wolbachia* strain showed genome expansion rather than the reduction that would be predicted by the symbiont genome reduction model.

Furthermore, the *w*Fol genome could contribute to understanding the genes and mechanisms underlying PI, as *w*Fol has been suggested to cause parthenogenesis in its host. All parthenogenetic *F. candida* lines carry *Wolbachia* whereas the sexually reproducing lines do not [25, 29, 30]. Unequivocal proof of PI would require curing of a parthenogenetic strain to produce males, but since *w*Fol induces parthenogenesis in addition to promoting host egg development, this experiment is not possible in the diplo-diploid *F. candida*. In several Hymenopteran species, *Wolbachia* has been shown to induce diploidy and feminization resulting in parthenogenetic reproduction [31, 32] and several draft genomes of PI-*Wolbachia* from Hymenopteran host are available: *w*Tpre from *Trichogramma pretiosum* and *w*Uni from *Muscidifurax uniraptor* [33–35]. Building a larger database of *Wolbachia* genomes associated with PI might shed more light on the genes involved in PI, similar to recent finding for CI [36].

In this study, we set out to explain the factors that contribute to the expansion in the *w*Fol genome, which can help further understand the genomic evolution within endosymbionts. Therefore, we updated and re-annotated the *w*Fol genome and compared it to a diverse set of high quality *Wolbachia* genomes. In addition, we searched for genes shared by *Wolbachia* associated with PI that could aid in understanding the mechanism behind this manipulation. To this end, we assembled a draft genome of an additional PI strain, *w*Lcla, that has been shown to cause diploidy restoration in the parasitoid wasp *Leptopilina clavipes* [31]. Finding the genes underlying PI in *Wolbachia* might elucidate the mechanism behind it and could resolve the debate on the nature of *w*Fol host interaction.

## Results

### Genome assemblies of *w*Fol and *w*Lcla

The short read corrected *w*Fol genome consisted of one contig with a total length of 1,801,626 bp and a GC-content of 34.35%. This was 43 bp longer than the initial assembly and the GC-content was a half percent higher [24]. The draft genome of *w*Lcla was assembled in 46 contigs with a total length of 1,150,755 bp and a GC content of 34.11%. Half of the assembly was contained in 9 contigs (L50) larger than 43,523 bp (N50). For a *Wolbachia* genome this is relatively small, but very similar in size to the other PI strain from supergroup B (*w*Tpre) [34]. However, *w*Tpre is in one scaffold that contains a total gap length of 16,680 bp and *w*Lcla is in 46 pieces, thus we still miss information for both of these genomes. Hence, the current assembly of *w*Lcla probably represents the major part the complete genome, but it will presumably increase slightly in size upon completion.

### Annotation of *w*Fol and *w*Lcla

To evaluate the quality and completeness of our annotations, we run the BUSCO pipeline based on 148 essential bacterial genes on the annotations of *w*Fol, *w*Lcla and an additional 16 *Wolbachia* annotations to compare with (see Additional file 1). The *w*Fol genome contained the most complete set of essential genes of the 18 *Wolbachia* analysed. It contained the complete sequences of 92.6% of the essential genes, including two duplications, and two fragmented. Only nine genes were missing, which was the lowest number of all *Wolbachia* analysed. The BUSCO gene set to which it was compared is a curated set of genes that are essential to most bacteria. However, in *Wolbachia* even complete genomes miss 9 to 25 genes from the BUSCO set, indicating that these genes probably are not missing from the assemblies and annotations but because they have become redundant for *Wolbachia* due to its endosymbiotic lifestyle. In the draft genome of *w*Lcla we found 133 complete (89.8%), two fragmented and 11 missing genes, which is very similar to most of the other *Wolbachia* genomes.

Annotating the improved *w*Fol genome uncovered a total of 1472 protein coding sequences (CDS), which is the largest number of CDS for any *Wolbachia* genome sequenced so far. This is not surprising given that it is the largest *Wolbachia* genome, and genome size and gene number correlate well in bacteria. However, a remarkably low number of pseudogenes were found in its genome. Only 2.8% of the genes appeared to be pseudogenized, while the pseudogene content in the other *Wolbachia* genomes ranged from 4.7 to 22.1%. In addition, we observed a high number of transposases, DNA-repair related genes and ankyrin repeat containing proteins (ANKs; see Table 1). The number of ANKs (96) and DNA-repair related genes (34) in *w*Fol outnumbered the quantity found in any other *Wolbachia* and the number of transposases is second only to the number found in *w*Cle.

**Table 1:** Genomic characteristics of 18 *Wolbachia* strains used for comparisons

Strain	Size (Mbp)	Contigs	CDS	Pseudo	Transposases	ANK	Repair	Resolvases
*w*Au	1.27	1	1204	62	119	39	18	4
*w*Bm	1.08	1	805	98	0	5	12	0
*w*Bol1-b	1.38	144	1139	162	11	2	23	2
*w*Cle	1.25	1	1216	NA	244	49	12	0
*w*Fol	1.80	1	1472	44	124	96	34	19
*w*Ha	1.30	1	1009	96	19	29	17	5
*w*Lcla	1.15	46	880	194	50	22	12	1
*w*Mel	1.27	1	1195	74	39	23	21	2
*w*No	1.30	1	1040	90	19	48	18	2
*w*Oo	0.96	1	647	195	0	3	7	0
*w*Ov	0.96	1	642	42	0	0	7	0
*w*Pip_Pel	1.48	2	1275	110	62	42	20	5
*w*Ppe	0.98	36	851	62	1	0	13	1
*w*Rec	1.12	43	902	127	26	2	13	1
*w*Ri	1.45	1	1150	114	67	29	18	4
*w*Tpre	1.13	9	1405	NA	53	54	16	2
*w*Uni	1.05	130	1174	NA	67	18	17	0
*w*VulC	1.66	10	1293	255	20	27	19	9

Column label abbreviations: CDS stands for coding sequences and ANK for ankyrin repeat containing protein. Annotations of *w*Cle, *w*Tpre and *w*Uni did not specify pseudogenes; for these strains this category is therefore labelled as not available (NA)

**Fig. 1:**
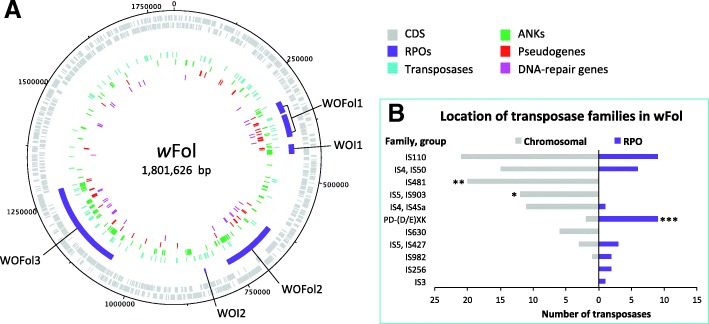
Distribution of genomic elements of interest in the *w*Fol genome. Legend: **a**) Circular map of *w*Fol displaying the distribution of genes of interest between regions of phage origin (RPOs) and chromosomal parts of the genome. Each ring gives locations of certain genes or genomic elements. From outer to inner ring: CDS forward strand, CDS reverse strand, RPO, transposases, ankyrin repeat containing genes (ANKs), pseudogenes and DNA-repair related genes. RPOs are separately labelled: WO regions with WOFol1 to 3 and WO-like islands with WOI1 and 2. **b**) Double side histogram that presents the distribution of transposase groups between the chromosomal and the RPO. Distributions were analysed with a binomial test in R, asterisks correlate to significant levels * to *p* > 0.05, ** to *p* > 0.01 and *** to *p* > 0.001

In addition, we found large numbers of phage genes, which were concentrated in five regions of phage origin (RPO) that were labelled WOFol1 to 3 and WO-like island 1 and 2 (see Additional file 2: Table S1). With a combined size of 458,452 base pairs, they took up 25.4% of the total genome size of *w*Fol, the largest amount in any *Wolbachia*. Remarkably, pseudogenes and the three before-mentioned overrepresented groups of genes (ANKs, DNA-repair related and transposases) were all unequally distributed between the phage regions and rest of the genome (see Fig. 1a). Of all groups more than the expected 25.4% was present within the RPOs, finding 29 out of 44 pseudogenes (binomial-test, R, *p* < 0.001), 23 out of 34 DNA-repair related genes (binomial-test, R, *p* < 0.001), and 42 out of 96 ANKs within them (binomial-test, R, *p* < 0.001). The distribution of transposases over the phage regions and rest of the genome was not different from a random expectation, since 26.6% of them were found in the RPOs (binomial-test, R, *p* = 0.75). However, when focussing on individual transposase families, they did show unequal distribution patterns (see Fig. 1b). While the IS4, IS110 and IS5 families were proportionally distributed over the whole genome, the PD-(D/E)XK family was more common in the RPOs than expected (binomial-test, R, p < 0.001). In addition, two of the other five families exclusive to either the RPOs or to the chromosomal part of the genome also showed a significantly skewed distribution (IS481: binomial-test, R, *p* < 0.01; IS5, IS903: binomial-test, R, *p* < 0.05).

The annotation of *w*Lcla uncovered 880 CDS and 194 pseudogenes (see Table 1). Thus, while it is similar in size to *w*Tpre, it contains 525 fewer CDS and 194 more pseudogenes. This is probably an effect of differences in annotation style, as no pseudogenes were annotated for *w*Tpre, but many truncated genes were found [34] that were therefore counted as one or two CDS instead of one pseudogene. Finally, *w*Lcla contained 4 phage-related genes as well as 7 phage-related pseudogenes, thus showing signatures of remnant phages. However, no conserved RPOs could be detected within the assembled contigs.

### Phage annotation and synteny

**Fig. 2:**
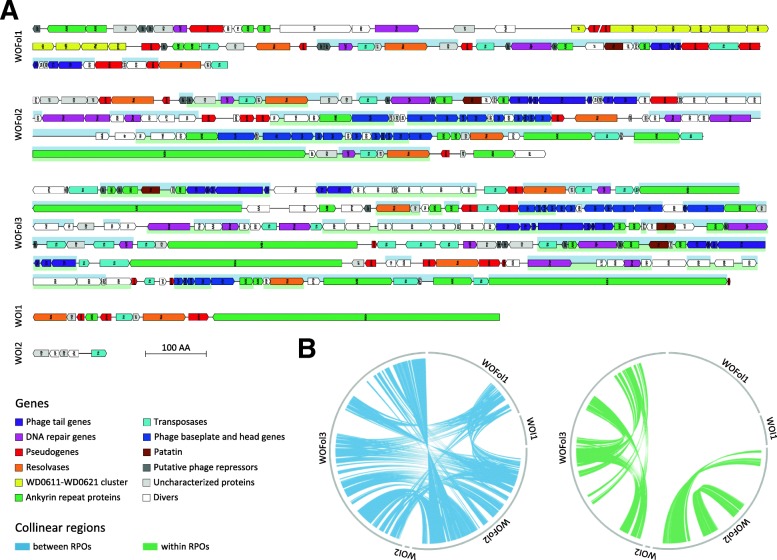
Detailed graphical overview of phage WO regions in *w*Fol. Legend: **a**) Graphical view of the RPO annotations of *w*Fol. Arrow blocks depict genes, upper marked regions are within collinear blocks between and lower marked regions within RPOs. A list of the microscopic abbreviations of gene annotations within the arrow blocks can be found in Additional file 3. **b**) Circular maps of collinear blocks between the RPOs of *w*Fol on the left (blue) and within (green) on the right

As the RPOs take up more than a quarter of the *w*Fol genome we put extra effort in their annotation. Kent et al. (2011) compared several WO phages and found that they are composed of multiple modules of genes that link to certain functions. We also found such modules in the WOFol phages, which included patatin-like phospholipases and baseplate, head and tail modules (see Fig. 2a: Additional file 3). However, not every module was present in all three WOFol regions, and WOFol2 and 3 contained many replicated modules. With a size of respectively 132.270 and 215.744 WOFol2 and 3 were the largest WO regions found in any *Wolbachia* (see Additional file 2: Table S1). Surprisingly, all three WOFol regions contained the tail module, which is often lost in other WO [37]. In addition, WOFol1 was missing the head and baseplate modules, but did contain the conserved WD0611-WD0621 cluster that is also found in several other WO phages [38]. All three WOFol regions also contained the recently described eukaryotic association module (EAM) [38] and within them 6 of the WO Latrotoxin CTD proteins. Further, the RPO of *w*Fol contained 19 resolvases, which is at least two times the number of resolvases found in any other *Wolbachia* genome (see Table 1). Resolvases are often connected to phage integration at specific sites, while integration by transposases is less site-specific [39]. Finally, WOFol 2 and 3 also harbored a bacteriophage abortive infection system, the AbiEi and AbiEii antitoxin-toxin complex, which has never been found in WO before. This system can provide phage protection at the population level by killing its host when infected by a new phage [40].

The large number of RPOs in *w*Fol led to the question if all these regions arose from a single ancestral phage that duplicated itself or, whether multiple phages infected the *w*Fol genome. Therefore, we searched for collinear blocks in the RPO and found them both between the three WOFol regions and within WOFol2 and 3 (see Fig. 2a, b). However, based on the mosaic structure of the collinear blocks there does not seem to be a complete duplication of one of the WO phages.

### Orthologue identification and orthogroup expansion

Orthofinder was run to cluster the complete set of 19,303 protein-coding sequences (CDS) of the 18 *Wolbachia* into orthogroups resulting in 1239 orthogroups containing 18,480 CDS (95.7%) (see Additional file 4: sheet Overall statistics). Of these, 460 were present in all *Wolbachia* genomes, including 414 that only contained single copy genes (see Additional file 4: sheet Orthogroups shared by all). These 460 genes can be considered the *Wolbachia* core genome, which is within the range of the 489 orthogroups found by Brown et al. (2016), when comparing members of supergroups A-D, F and L. For *w*Fol, 1346 of the 1472 CDS were grouped into 936 of the orthogroups (see Additional file 4: sheet All orthogroups). Thus, 126 CDS remained unassigned and were therefore considered to be unique to *w*Fol (see Additional file 4: sheet Unassigned genes). *w*Tpre was the only genome containing more unique CDS. This was most likely due to the many truncated/pseudogenes in this genome annotation [34], which probably have ended up in separate groups because of Orthofinder’s algorithm sensitivity for gene length [41], thus creating false orthogroups of truncated genes. Most of the unassigned genes from *w*Fol were hypothetical proteins (76) and putative membrane proteins (27), but 23 of these genes could be annotated in more detail (see Additional file 4: sheet Unassigned genes). Moreover, 35 (27.8%) of the unassigned CDS were located within the RPOs, which include the AbiEi and AbiEii antitoxin-toxin complex mentioned above (Additional file 5). For the *w*Lcla draft genome, 875 of their 879 CDS were grouped into 817 orthogroups, while only 4 remained unassigned. Three of these were annotated as hypothetical proteins and one was a transposase (see Additional file 4: sheet Unassigned genes). Therefore, no distinct functions could be linked to these genes.

**Fig. 3:**
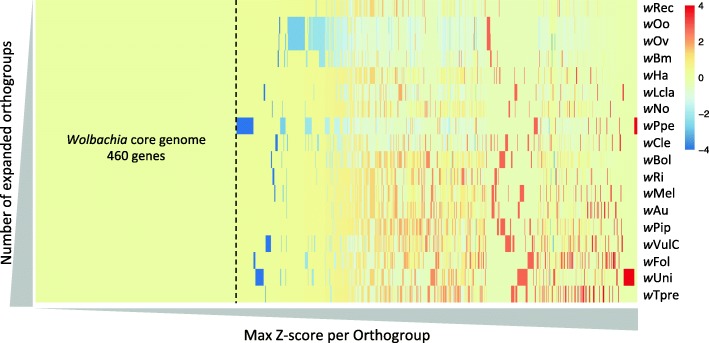
Expanded and contracted orthogroups in different *Wolbachia* strains. Legend: Heatmap of the Z-scores of the 18 *Wolbachia* strains per orthogroup that indicates expansions in red and contractions in blue. The rectangle separated by a dashed line represents the 460 core genome genes without variation in Z-score. *Wolbachia* strains are ordered by the number of expanded orthogroups from the least on top till the most at the bottom and all orthogroups are sorted on their max Z-score from lowest at the left to highest at the right

To define orthogroup expansions and contractions, Z-scores were calculated for each orthogroup (see Fig. 3 and Additional file 4: sheet all orthogroups). Z-scores measure the deviation of the number of genes of one strain from the average number of genes per strain in an orthogroup. Orthogroup expansions had more genes (Z-score 2 or higher) and contractions fewer (Z-score − 2 or lower).

The three genomes with the highest number of gene family expansions were all linked to PI (*w*Tpre, *w*Uni and *w*Fol). Unfortunately, no orthogroup was expanded in all three *Wolbachia* genomes. Focussing on the *w*Fol genome we found 72 expanded and 10 contracted orthogroups (see Additional file 4: sheet Expanded or contracted in *w*Fol). All 10 contracted orthogroups were genes completely missing in *w*Fol. The 72 expanded orthogroups contained 371 genes of which more than half within the RPOs (194 genes within 21 orthogroups). In line with this, 89 genes were phage-related genes, pressing the notion that the expansion of *w*Fol is for a large part due to an increase of phage genes. In addition, more than half (190) of the genes were transposases (93), ANKs (74) and DNA-repair related (21), proving that these groups are overrepresented in this genome.

Not surprisingly, considering its small size the *w*Lcla genome contained only 14 expanded orthogroups with 26 genes in total (see Additional file 4: sheet Expanded or contracted in *w*Lcla). Eight of these genes were within one orthogroup of IS110 family transposases, while all other groups consisted of 1 gene missing in all other *Wolbachia* or 2 duplicate genes in *w*Lcla. Most of these other groups consisted of uncharacterized proteins without a clear function and the genes with annotation had diverse functions in several biological processes. The *w*Lcla genome contained only five significantly contracted orthogroups, which is surprising given the large number of pseudogenes. This indicates that many of the pseudogenized genes of *w*Lcla were also degraded in other *Wolbachia.*

### Parthenogenesis induction genes

**Fig. 4:**
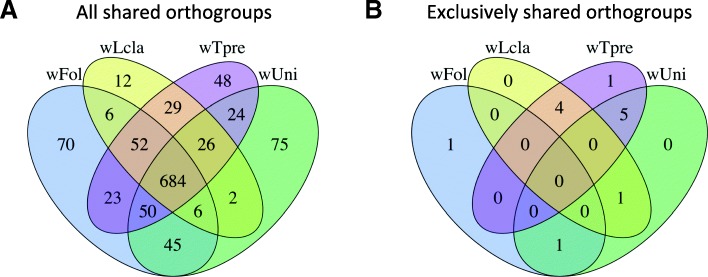
Orthogroups shared by PI *Wolbachia.* Legend: Venn diagrams representing **a**) a distribution of all orthogroups shared between the PI associated *Wolbachia*, including the orthogroups shared with the other 14 *Wolbachia* and **b**) a distribution of all orthogroups exclusively present in the PI associated but not in the other 14 *Wolbachia*

Clustering all genes into orthogroups also allowed us to search for orthogroups whose absence or presence was associated with the four PI-*Wolbachia* strains (*w*Fol, *w*Lcla, *w*Tpre and *w*Uni). We found no genes that were exclusively missing from the four PI-*Wolbachia*. There was a large number of shared orthogroups (684) between the four genomes, which obviously also included the 460 core genome orthogroups (see Fig. 4a). However, there were no orthogroups that were exclusively present in all four or three out of the four parthenogenesis-associated *Wolbachia* genomes (see Fig. 4b). Only between pairs of the PI-*Wolbachia* 11 exclusive orthogroups existed (see Additional file 4: sheet Exclusive or mainly in PI). Five of those contained only uncharacterised proteins and another two contained only transposases. The remaining four consisted of: 1) a putative phage protein shared by *w*Uni and *w*Fol, 2) a protein with a magnesium transported domain shared between *w*Tpre and *w*Uni and 3) a cluster of two genes shared between *w*Tpre and *w*Lcla consisted of a protein with a partial fungal domain of unknown function and a protein that contains several leucine-rich repeats. These leucine-rich repeats are protein binding domains that are involved in a wide range of biological processes [42]. Furthermore, we also searched for orthogroups that were present in at least three of the four PI strains and in a maximum of two other *Wolbachia* strains*.* This yielded two orthogroups with single copy uncharacterized genes, represented in *w*Fol by wFol_04740 and wFol_12640, and both shared by *w*Fol, *w*Lcla, and *w*Tpre (see Additional file 4: sheet Exclusive or mainly in PI). Absence of these genes in *w*Uni strain might be inaccurate, as the still quite fragmented assembly is possibly hampering gene annotation (Table 1). One orthogroup was also found in the male-killing strain *w*Bolb-1 and the other orthogroup in both *w*Bolb-1 and CI-strain *w*Pip_Pel. Based on their annotation neither of the examined genes could be directly linked to parthenogenesis induction.

### Whole genome correlations

**Fig. 5:**
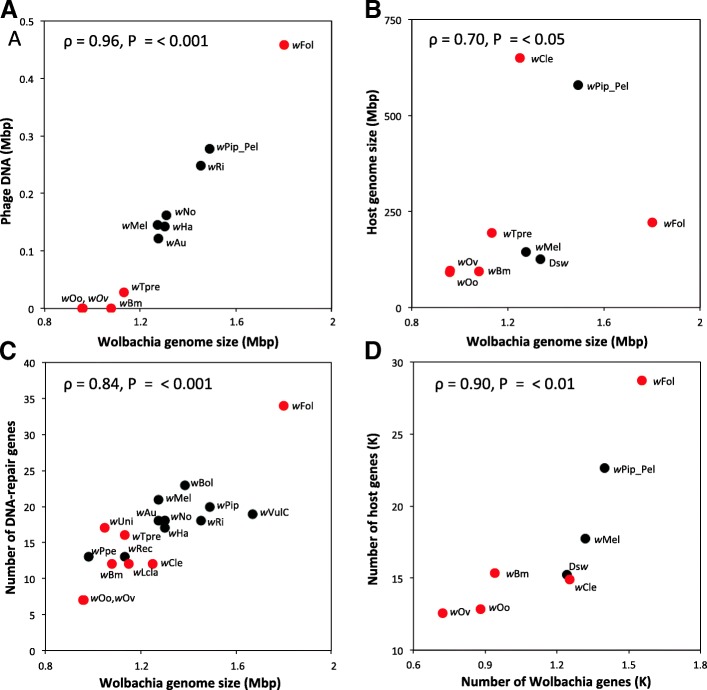
Multiple positive correlations associated with genome evolution in *Wolbachia.* Legend: Correlations between **a**) genome size and amount of integrated phage DNA both in number of basepairs, **b**) host and *Wolbachia* genome size in mega basepairs, **c**) number of DNA-repair related genes and *Wolbachia* genome size and **d**) host and *Wolbachia* number of genes. Red data points indicate obligate transitional and black facultative endosymbionts; all points are labelled with strain names and for the correlations with the host the four strains residing in *Drosophila simulans* were averaged and labeled as Ds*w*. Spearman’s rank correlations and corresponding *P*-values calculated in R are shown in the top corner of every plot

We found a positive correlation between *Wolbachia* genome size and the amount of phage DNA it contained (see Fig. 5a). This indicates that a significant portion of the variation in genome size was accounted for by the RPOs. The genomes of *Wolbachia* strains that are essential to their host occurred at both ends of the size spectrum, with most having small genomes and little phage DNA and *w*Fol having a large, phage-rich genome. In addition, we also found a positive correlation between the number of DNA-repair related genes and the genome size of *Wolbachia* (see Fig. 5b)*.*

Host genome size and gene number were also correlated to *Wolbachia* genome size and gene number, respectively (see Fig. 5c, d), with gene content being more strongly correlated. Moreover, the correlation between genome sizes still holds when RPO are excluded (ρ = 0.86, *P* = 0.01; see Additional file 6), although the deviating strain *w*Cle needed to be excluded as the RPO could not be properly annotated and therefore their size could not be determined. These correlations suggest that the genome size and number of genes of a *Wolbachia* are not independent from the size and number of genes of its host genome.

## Discussion

The *w*Fol genome is the largest *Wolbachia* genome to date, and our results suggest that its genome size has increased due to the integration of several phages, as phage regions take up more than a quarter of the total *w*Fol genome. This is supported by an expansion of 21 orthogroups containing phage genes. These regions of phage origin (RPOs) had higher numbers of ankyrin repeat containing genes (ANKs), DNA-repair related genes, pseudogenes and PD-(D/E)XK transposases. In addition, they contained a bacteriophage abortive infection system new to *Wolbachia*. Comparing *w*Fol, *w*Lcla and two other PI-associated strains to a diverse set of 14 *Wolbachia* genomes did not elucidate a set of genes unique to the four PI strains. However, there were unique sets of genes between some pairs of the PI associated strains. Finally, we found strong correlations between *Wolbachia* and host gene numbers, suggesting that there might be convergent evolution between *Wolbachia* and their host genomes.

### Genome expansion in *w*Fol

The first question we set out to answer was why the genome of the obligate *Wolbachia* endosymbiont of the parthenogenetic springtail *F. candida* (*w*Fol) has expanded, while current theory predicts an obligate relationship to lead to a reduced genome size [12, 18]. Obligate endosymbionts usually have a small genome size and contain few repeated and mobile elements such as transposases [43], ANKs [44, 45] and RPOs [46, 47]. Genome reduction of this kind is also evident in the genomes of obligate *Wolbachia* strains in filarial nematodes (*w*Oo, *w*Ov and *w*Bm), which are the smallest complete *Wolbachia* genomes, containing no or very few transposable elements and phage derived genes [9, 10, 48]. In contrast, the genome of the obligate *w*Fol strain is the largest complete *Wolbachia* genome discovered to date with a length of 1,801,626 base pairs (bp) [24]. Moreover, upon annotating this unusually large genome meticulously, we found that it is highly enriched in repeated and mobile elements and that the RPOs take up more than a quarter of its genome. These regions contain many of the ANKs and transposases that are enriching this genome and have a big influence on the genomic structure and size of *w*Fol.

Many expanded orthogroups were also found to be located in the RPO, including a set of genes connected to DNA repair. Integrated phage genes within endosymbionts can still be expressed and are known to influence bacterial and host processes [36, 49]. Therefore, the functions of these integrated genes may affect the performance of the tri-partite symbiosis. Typically, in obligate endosymbiont genomes a depletion of DNA repair genes is seen and the loss of these genes would result in a higher effective mutation rate [13]. Thus, the gain in DNA repair genes in the *w*Fol genome can be expected to lower effective mutation rate, resulting in a better-conserved genome with fewer deletions and fewer pseudogenized genes.

We also found a large number of unique genes present in *w*Fol. Although RPOs are known to be a source of new genes [38, 45, 50], in *w*Fol unique genes were evenly distributed over the genome. A possible explanation for the large number of novel genes could be that *w*Fol is the first annotated *Wolbachia* genome of supergroup E, while many of the other supergroups were represented with more genomes in our analysis. Therefore, this group of unique genes might not just represent genes unique to *w*Fol but also include genes that are specific to the entire E supergroup. Nevertheless, the presence of this many unique genes indicates that either *w*Fol specifically or all members of supergroup E are able to acquire new genes or preserve existing ones more easily compared to other *Wolbachia*.

Another strong signature of gene conservation is the low number of pseudogenes found in *w*Fol. Most of the pseudogenes that were present were located within the RPO, suggesting a less stringent conservation of phage-derived genes. This is in line with what is seen in other *Wolbachia* where the phage regions are often more prone to degradation [51]. However, the rather high share of intact phage genes within *w*Fol, suggest that even these regions with higher degradation rates are still being maintained at an elevated rate. The combined results on phage preservation, the low number of pseudogenes, the many DNA repair genes, and the large number of unique genes consistently point towards a genome shaped by gene preservation. This is inconsistent with expectations based on the symbiont genome reduction model.

At first glance, the *w*Fol genome with its relatively large size and many mobile elements resembles a recently host-restricted symbiont. However, these symbionts are generally facultative for their host, harbour many pseudogenes and suffer large and small deletions, as their genomes change rapidly [12, 18]. By contrast, *w*Fol is obligate to its host, possesses only few pseudogenes, and shows no signs of large deletions. Furthermore, the start of the symbiosis between *w*Fol and its host probably coincided with the host becoming parthenogenetic. Although, this split has not been properly dated yet, genetic evidence shows considerable differentiation between sexual and parthenogenetic lines of *Folsomia candida* [29], suggesting this was not a recent event. Thus, *w*Fol does not fit the description of a reduced long-term obligate symbiont genome, nor a recently host-restricted symbiont genome. Rather, it seems to fall into a new category of a long-term obligate symbiont that was able to avoid genome degradation.

This leads to the question: what has caused the genome preservation in *w*Fol compared to the other *Wolbachia*, including the other PI and obligate strains? These obligate strains are just like *w*Fol vertically transmitted within hosts that are dependent on their *Wolbachia*, thus there must be another explanation for the differences in genome maintenance than level of interdependence with their host or form of transmission. Interestingly, there are unique features to the genome of *F. candida*, which mirror the genomic patterns of its symbiont. Its genome has with 28,734 genes the most gene rich genome of the *Wolbachia* hosts (Fig. 5d). Moreover, it is also gene richer than the two other published Collembolan genomes of *Holacanthella duospinosa* where they found 12,000 gene models [52] and *Orchesella cincta* with 20,249 genes [53]. In addition, groups of genes related to DNA repair expanded in both genomes, with *F. candida* having expanded groups of ATP-dependent DNA helicases which are important for DNA repair [24, 54]. Finally, both host and endosymbiont contain high amounts of foreign DNA, as 2.8% of *F. candida* genes are horizontally transferred genes (HTG) from a wide variety of organisms including bacteria, fungi and protists, but not from *Wolbachia*. This might sound low compared to the 25.4% of phage DNA in *w*Fol but is in fact the highest percentage found in any metazoan genome except for rotifers and some nematode species [24] Moreover, this abundance of mostly intact HTG was correlated with an increase in transposons. Thus, also the *F. candida* genome seems focused on preserving genes. This is as far as we know, the first case that shows signs of convergent evolution between endosymbiont and host genomes. In addition, the correlation between *Wolbachia* gene number and host gene number of all combinations with usable genomes, suggest that convergence between host and endosymbiont genomes may have taken place in more *Wolbachia* strains.

### Regions of phage origin

The integration of phage DNA is one of the main reasons for the large size of the *w*Fol genome. The RPO included three phage WO regions and two phage WO-like islands, which is within the range of two to five prophage WO haplotypes found in other fully sequenced Wolbachia genomes [37]. However, the size of the *w*Fol WO regions is much larger and two of the regions contain multiple copies of essential phage gene clusters. Possibly, multiple phages clumped together within the *w*Fol genome or the phages that integrated had multiple copies of the same modules. Alternatively, a recent duplication took place but this is not very likely because the collinear blocks between phages were very fragmented and the longer blocks contained mainly the conserved modules found in all WO phages. Such large clusters containing multiple sets of the same phage modules have previously been found in *Wolbachia* genomes *w*Pip and *w*No [50, 55] and hamper an assessment of the exact number of phages that integrated within this genome.

All three WO regions within *w*Fol contained the characteristic elements and standard modules of phage WO. These included the Patatin gene that is probably helpful for entering the host cell [37] and the recently defined eukaryotic association module including latrotoxin-CTD domain proteins, which are related to black widow venom genes [38]. Neither of the WO regions contained the *cifA* and *cifB* genes linked to CI [36], therefore it is very unlikely that *w*Fol can cause CI. Some *w*Fol RPO features are exceptional, such as the vast amount of resolvases and transposases, which are thought to be involved with phage integration into their bacterial host [39, 56]. However, integration by transposases usually takes place at non-specific integration sites while resolvases use conserved sites [39]. Thus, the excessive presence of both transposases and resolvases in the *w*Fol phages raises questions concerning their mode of integration. Finally, a toxin-antitoxin system that is new for *Wolbachia* was found in the WOFol2 and 3. This AbiEi and AbiEii system is an abortive infection system that can cause altruistic cell death to prevent new phages from settling within the bacterial population [40]. One might argue that such a system may prevent further genome expansions by additional phages.

### Genetic basis of parthenogenesis induction

We also perused the *w*Fol genome for genes that are associated with PI to elucidate the genetic basis of this reproductive manipulation. Together with three other PI *Wolbachia*, *w*Uni of supergroup A and *w*Tpre and *w*Lcla of supergroup B, they were compared to a diverse set of 14 *Wolbachia* including members of 6 different supergroups (A,B,C,D,F and L). We searched for gene sets present in at least three PI and a maximum of two other *Wolbachia* strains, comparable to the CI phenotype where a toxin-antitoxin gene cluster was shared by all CI inducing or repressing strains and some others [36, 57]. We did not find any genes present in all PI strains, which may have several non-mutually exclusive explanations. First, PI genes may not be uniquely present in *Wolbachia* expressing the PI phenotype, but also in some of the *Wolbachia* causing other reproductive manipulations, similar to what is seen for the CI phenotype [58]. The two genes we identified to be associated with three of the PI-strains were also present in *w*Bolb-1 and in *w*Pip_Pel that are both in supergroup B. The presence of these genes in the two other strains might have to do with the fact that phenotypic expression of *Wolbachia* has been found to be determined in combination with the host genotype. For example, the CI-strain *w*Rec caused male killing after being transferred to a new host [59] and *w*Tei caused CI after a host transfer while showing no signs of reproductive manipulation in its natural host [60]*.* This same interaction with host genotype may apply to PI expression. A second possibility is that the PI phenotype is not a single genotype but is achieved via different cellular mechanisms formed by convergent evolution, in which case we do not expect a single gene cluster to be shared by all PI-*Wolbachia*. Support for this explanation can be found in studies showing that parthenogenesis is induced in a two-step mechanism of diploidization of the gamete followed by feminization of the egg [61, 62]. Diploidization can be achieved in different ways. In both *Trichogramma* wasps and *Leptopilina clavipes* (the hosts of respectively *w*Tpre and *w*Lcla, the two PI inducing *Wolbachia* of supergroup B) gametes are rendered diploid by failure of the first mitotic anaphase division [31, 63]. In *Muscidifurax uniraptor* (host of *w*Uni of supergroup A) diploidization is only restored after the second mitotic division through a fusion of the two mitotic nuclei [64]. For, *w*Fol of supergroup E the speculated mechanism is diploidy restoration through either non-disjunction or fusion of the two haploid division products at the end of the first anaphase [65] and thus similar to the situation in *w*Tpre and *w*Lcla, although the difference in sex determination system might call for a different manipulation for the same outcome. Therefore it has already been suggested that diploidy restoration in *Wolbachia* most likely has a polyphyletic origin [31]. With this in mind, the cluster of two genes uniquely shared by *w*Tpre and *w*Lcla might still be very interesting and might be linked to one of the two steps of PI in these lines that seem to have a similar mechanism developed in supergroup B.

## Conclusions

Large RPOs with ample repair genes and accumulation of repetitive and transposable elements make up most of the expansion of the *w*Fol genome. This genomic signature of gene conservation was mirrored in the *F. candida* host genome. We found that a large part of the variation within the genome size and gene number of facultative endosymbionts is correlated to the gene number of the host. This suggests that host and symbiont genome may be subject to correlated selection pressures that resulted in convergent evolution between host and endosymbiont, or that somehow the host may have a direct influence on the symbiont genomes. However, these selection pressures would probably be neutralized after endosymbionts move into a specialized cell, explaining the well-documented steady genome decrease seen in those cases [11, 66, 67]. Therefore, genome reduction may ultimately result from becoming obligate. However, before the endosymbiont resides in a specialized cell, but while being mainly transmitted vertically, genome expansion of endosymbiont genomes might occur.

The search for the PI genes yielded a set of potential candidate genes. Elaborating on these findings could answer whether the PI genes are monophyletic or polyphyletic. The complexity of this trait and the indications that this is caused by multiple genes could also means that both are not mutually exclusive in this case.

## Methods

### Assembling of *w*Fol and *w*Lcla

Sequencing and assembling of the *w*Fol genome was described in Faddeeva-Vakhrusheva et al. (2017). We corrected this assembly using the Illumina HiSeq 2000 data of Gerth et al. (2014) (NCBI accession number: SRR1222159). Illumina reads were mapped to the assembly with BWA [68] using default parameters. Variants between the assembly and the mapped Illumina reads were called and quality filtered using GATK v. 3.7 [69], filtered based on read depth with vcffilter (“DP > 10”) of the Vcflib package (E. Garrison, 2012, https://github.com/vcflib/vcflib). Variants were inspected manually and when they were supported by the mapped Illumina data they were included in the genomic sequence.

Reads used for the *w*Lcla assembly were taken from a study that sequenced its host *Leptopilina clavipes* [70] using both an Illumina HiSeq (HiSeq) and a Pacific Biosciences RS I (PacBio) library. Hiseq reads were used to error correct the PacBio reads with the PacBioToCA pipeline of CeleraAssembler7.0 [71]. We used the corrected PacBio reads for the *w*Lcla assembly. To filter out the *Wolbachia* reads, all PacBio reads were mapped onto the *w*Tpre and the *w*Pip_Pel genome with BLASR [72]. A consensus based on the mapped reads was made per reference genome with PBDAG-Con [73] and the resulting contigs were extended and joined with PBjelly [74]. Thereafter, all reads were mapped back to these preliminary assemblies and PBDAG-Con and PBjelly were run again to extend the preliminary contigs further. All reads mapping to these two assemblies were extracted and assembled de novo in Mira [75] with the following parameters: COMMON_SETTINGS –SK:mmhr = 1 PCBIOHQ_SETTINGS –CO:mrpg = 5. Next, all corrected Pacbio reads were mapped back to the Mira assembly, after which two iterations of the combination of PBDAG-Con and PBjelly were run to extend and connect the contigs as much as possible. The process ended with a final step of PBDAG-Con to confirm the extensions based on mapped reads. Finally, the HiSeq data was mapped to the assembly, variants were called, manually checked and the assembly was adjusted in the same way as described for the *w*Fol assembly.

### Annotation of *w*Fol and *w*Lcla

The annotation of both genomes was done using the DIYA pipeline [76], in which we included: Prodigal for gene prediction [77], tRNAscan-SE and RNAmmer to predict RNA features [78, 79] and GenePRIMP to mark possible pseudogenes and short genes without annotation [80]. Predicted genes smaller than 100 amino acids without hits in any of the databases were removed. Genes were manually annotated as pseudogenes when they contained frameshift mutations, premature stop-codons, missing start codons or disruptions by IS-insertions. In addition, protein domains were predicted according to the Pfam database [81], BlastP to the NCBI database [82] and FASTA searches [83] against a well-curated in-house database by Lisa Klasson of *Wolbachia* genomes. All results were loaded into Artemis [84] in which they were manually curated. Transposases were blasted to the IS-finder database to determine the family and group (https://www-is.biotoul.fr). Uncharacterized/unique proteins were run through the InterPro databases with InterProScan [85]. Genes that did not exhibit features indicative of any specific function, but did contain transmembrane segments, cytoplasmic and non-cytoplasmic domains were characterised as putative membrane proteins.

For *w*Fol, equal distribution of gene groups and transposase families over chromosomal parts (74.6%) and RPOs (25.4%) was tested with binomial tests in R [86].

### Annotation completeness and ortholog identification

We compared the *w*Lcla and *w*Fol assemblies to a set of 16 other *Wolbachia* genomes, selected based on assembly quality as well as phylogenetic and functional diversity (Additional file 2: Table S2) [9, 10, 28, 33–35, 50, 51, 55, 87–92]. Protein sequences of these 16 genomes were downloaded from the NCBI database and the completeness of their gene content was predicted with the BUSCO v3 pipeline, which compares the *Wolbachia* genomes to a set of 148 single copy bacterial genes (Bacteria odb9) [93]. Furthermore, orthogroups were inferred using OrthoFinder and are defined as groups of genes that all derived from a single gene in the last common ancestor [41]. This allowed us to look for orthogroups shared between *Wolbachia* associated with PI and find unique genes (genes lacking orthologues in other *Wolbachia*).

In all genomes, expanded and contracted orthogroups were identified by calculating the z-scores [94], which is calculated by subtracting the average number of genes in an orthogroup over all species from the gene number of a focal species and dividing this by the standard deviation. A z-score of 2 or above indicates an expansion and a score of − 2 or below a contraction of an orthogroup.

### Prophage annotation and synteny

Prophage regions of *w*Fol and *w*Lcla were initially identified by PHASTER [95]. While this correctly finds the more common phage genes it does not identify the more diverse EAM [38], as they contain of eukaryotic genes picked up by phages that are therefore not being recognised as phage genes. Therefore, the EAM were defined during manual curating of the prophage regions. Phage region lengths of other phages were based on annotations from Bordenstein & Bordenstein (2016) to include the whole phages with EAM or, if not available from that paper, by determining them in a similar way (Additional file 2: Table S1). As, the phage region are often the most difficult parts to assemble, this was only done for *Wolbachia* assemblies with two or less scaffolds, to avoid the use of incomplete sets of RPOs within our analysis. Moreover, *w*Cle could not be used as phage genes seemed to be removed from its annotation.

Collinearity between the regions of phage origin (RPOs) was analysed with the MCScanX package [96]. A BlastP search of all against all RPO protein sequences was performed with an E-value cutoff of 1e− 10, followed by a MCScanX run (−m 2). Synteny plots were drawn using Circos [97].

### Whole genome correlations

To examine overall genomic expansion and contraction patterns and their possible connection to phages and their host, we assessed correlations between *Wolbachia* genome size versus amount of phage DNA, *Wolbachia* genome size versus host genome size, and number of *Wolbachia* genes versus number of host genes for all combinations that had sufficient reliable data. Genomic information of all available hosts of the *Wolbachia* analysed in this study was collected from online servers, most were downloaded from NCBI (hosts and accession numbers: *Brugia malayi,* GCA_000002995.4; *Cimex lectularius,* GCF_000648675.1; *Culex quinquefasciatus,* GCA_000209185.1; *Drosophila melanogaster,* GCF_000001215.4; *Drosophila simulans,* GCA_000754195.2; *Folsomia candida,* GCA_002217175.1; *Onchocerca ochengi,* GCA_000950515.2 and *Trichogramma pretiosum*, GCF_000599845.1) and data for *Onchocerca volvulus* strain Cameroon was collected from http://parasite.wormbase.org/Onchocerca_volvulus_prjeb513/Info/Index/. Spearman’s rank correlations were calculated in R.

## Additional files

Additional file 1: BUSCO assessment results of 18 *Wolbachia* genomes included in this paper. Protein sequences of the 18 *Wolbachia* strains were searched for a set of 148 single copy bacterial genes (Bacteria odb9), defining the complete, fragmented and missing genes. (PDF 291 kb)

Additional file 2: Supplementary tables on *Wolbachia* strains. **Table S1.** on RPO regions within the *Wolbachia* strains analysed and **Table S2.** with extra information on these strains. (DOCX 92 kb)

Additional file 3: Abbreviations Fig. 2. List of abbreviations of annotations within arrows in Fig. 2. (DOCX 96 kb)

Additional file 4: Results OrthoFinder analysis. This file contains multiple sheets with selected orthogroups derived from the Orthofinder analysis as well as the z-scores. (XLSX 1245 kb)

Additional file 5: Distribution of unassigned *w*Fol genes. Double sided histogram that presents the distribution of unique *w*Fol genes between the chromosomal and the regions of phage origin (RPO). (PDF 34 kb)

Additional file 6: Host genome size to *Wolbachia* genome size excluding RPOs. Correlation between host genome size and *Wolbachia* genome size excluding RPOs. Red data points indicate obligate transitional and black facultative endosymbionts; all points are labelled with strain names and for the correlations with the host the four strains residing in *Drosophila simulans* were averaged and labeled as Ds*w*. (PDF 36 kb)

Additional file 7: *w*Fol annotation. GenBank data file with the *w*Fol annotation described in this paper. (GBK 2967 kb)

Additional file 8: *w*Lcla annotation. GenBank data file with the *w*Lcla annotation described in this paper. (GBK 2292 kb)

## Abbreviations

ANK: Ankyrin repeat containing protein
CDS: Protein coding sequences
CI: Cytoplasmic incompatibility
EAM: Eukaryotic association module
PI: Parthenogenesis induction
RPO: Region of phage origin
