# Supplementary figures and images for: Genome expansion of an obligate parthenogenesis-associated Wolbachia poses an exception to the symbiont reduction model
Source: BMC Genomics. 2019 Feb 6;20:106. doi: 10.1186/s12864-019-5492-9 (PMC6364476; doi:10.1186/s12864-019-5492-9)

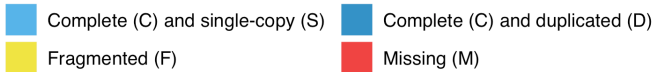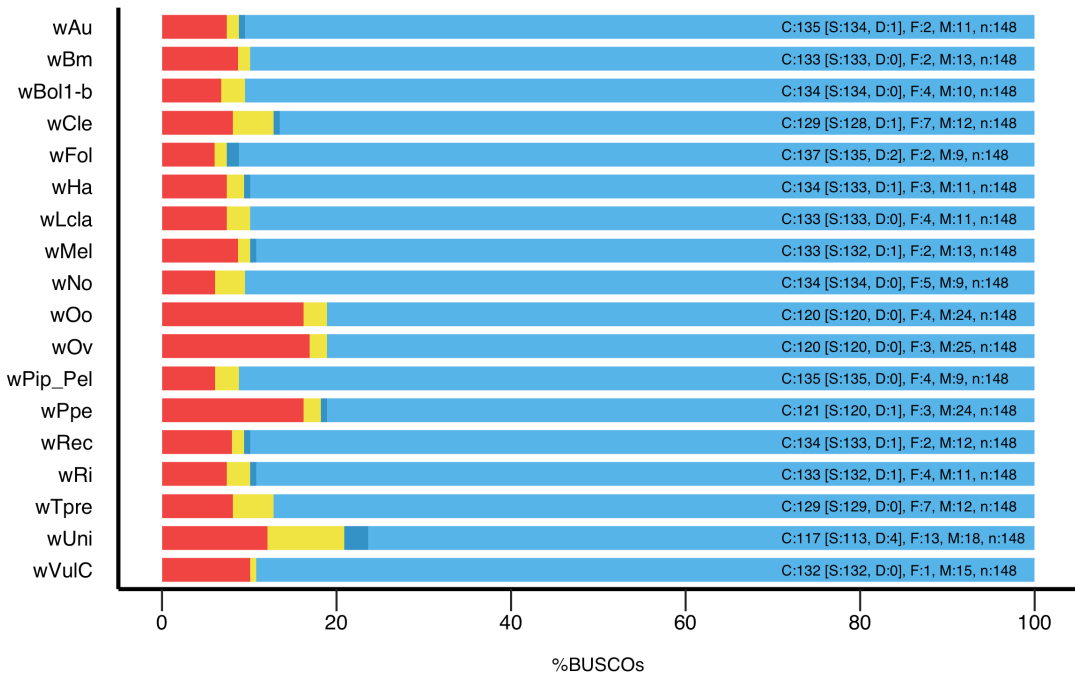

Supplement: Supplementary file 1 — BUSCO assessment results of 18 Wolbachia genomes included in this paper. Protein sequences of the 18 Wolbachia strains were searched for a set of 148 single copy bacterial genes (Bacteria odb9), defining the complete, fragmented and missing genes. (PDF 291 kb) [file 12864_2019_5492_MOESM1_ESM.pdf]

■ Chromosomal (N = 91) ■ RPO (N = 35)

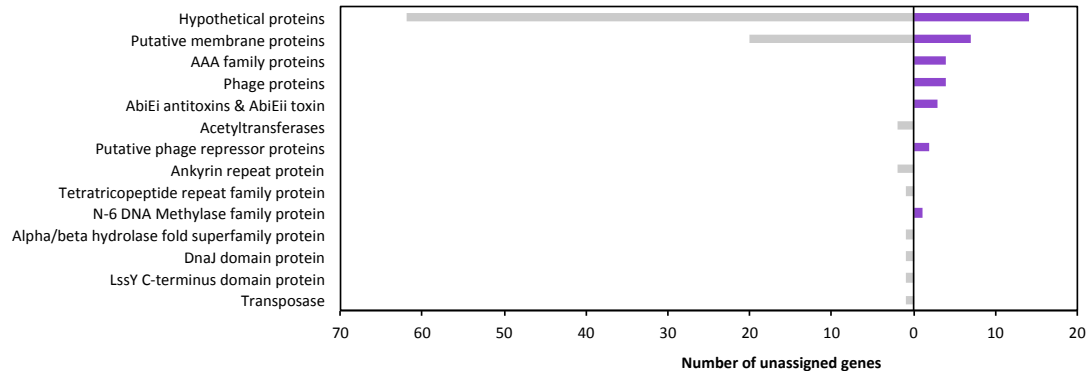

Supplement: Supplementary file 5 — Distribution of unassigned wFol genes. Double sided histogram that presents the distribution of unique wFol genes between the chromosomal and the regions of phage origin (RPO). (PDF 34 kb) [file 12864_2019_5492_MOESM5_ESM.pdf]

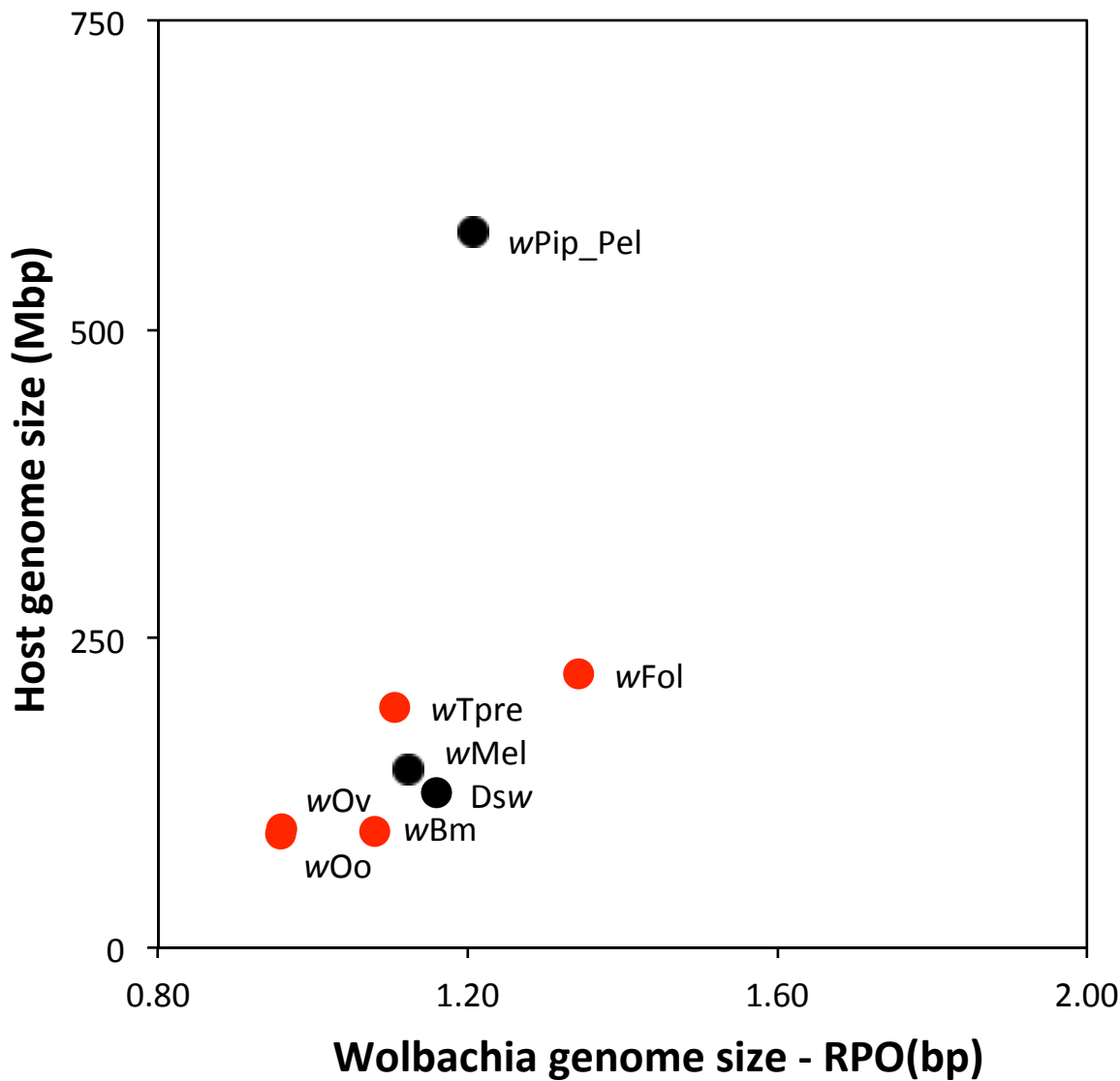

Supplement: Supplementary file 6 — Host genome size to Wolbachia genome size excluding RPOs. Correlation between host genome size and Wolbachia genome size excluding RPOs. Red data points indicate obligate transitional and black facultative endosymbionts; all points are labelled with strain names and for the correlations with the host the four strains residing in Drosophila simulans were averaged and labeled as Dsw. (PDF 36 kb) [file 12864_2019_5492_MOESM6_ESM.pdf]
