# Supplementary material for: Genome expansion of an obligate parthenogenesis-associated Wolbachia poses an exception to the symbiont reduction model
Source: BMC Genomics. 2019 Feb 6;20:106. doi: 10.1186/s12864-019-5492-9 (PMC6364476; doi:10.1186/s12864-019-5492-9)
Supplement: Supplementary file 2 — Supplementary tables on Wolbachia strains. Table S1. on RPO regions within the Wolbachia strains analysed and Table S2. with extra information on these strains. (DOCX 92 kb) [file 12864_2019_5492_MOESM2_ESM.docx]

**Supplementary table 1**: Characteristics of RPOs within the *Wolbachia* strains used in this study with fewer than three scaffolds and properly annotated phage genes.

| **Wolbachia** | **Phage** | **Locus-tags** | **Length (bp)** | **Total (bp)** |
| --- | --- | --- | --- | --- |
| *w*Au | WOAu1-2 | WPAU_0243-WPAU_0329 | 67.918 | 121.654 |
|  | WOAu3 | WPAU_0623-WPAU_0672 | 53.736 |  |
| *w*Bm strain TRS | NA | NA | NA | NA |
| *w*Fol | WOFol1 | wFol_02640-wFol_03410 | 83.672 | 458.452 |
|  | WOFol2 | wFol_05200-wFol_06400 | 132.270 |  |
|  | WOFol3 | wFol_08880-wFol_10640 | 215.744 |  |
|  | WO-Island 1 | wFol_03590-wFol_03680 | 23.233 |  |
|  | WO-Island 2 | wFol_06910-wFol_06950 | 3.533 |  |
| *w*Ha | WOHa1 | wHa_02010-wHa_02760 | 100.443 | 142.542 |
|  | WOHa2 | wHa_03390-wHa_03840 | 42.099 |  |
| *w*Mel | WOMelA | WD0253-WD0296 | 38.175 | 144.430 |
|  | WOMelB | WD0563-WD0647 | 87.451 |  |
|  | WO-Island | WD0507-WD0514 | 18.804 |  |
| *w*No | WONo1 | wNo_01000-wNo_01400 | 41.397 | 161.733 |
|  | WONo2 | wNo_07170-wNo_07380 | 13.585 |  |
|  | WONo3 | wNo_09000-wNo_09150 | 21.354 |  |
|  | WONo4 | wNo_10070-wNo_10350 | 47.238 |  |
|  | WO-Island 1 | wNo_01980-wNo_02200 | 31.868 |  |
|  | WO-Island 2 | wNo_05070-wNo_05150 | 6.291 |  |
| *w*Oo | NA | NA | NA | NA |
| *w*Ov | NA | NA | NA | NA |
| *w*Pip_Pel | WOPip1-3 | WP0236-WP0354 | 129.469 | 277.399 |
|  | WOPip4 | WP0405-WP0465 | 71.818 |  |
|  | WOPip5 | WP1289-WP1352 | 76.112 |  |
| *w*Ri | WORiA | wRi_012450-wRi_012680 | 22.148 | 248.301 |
|  | WORiB | wRi_005310-wRi_005930 | 73.575 |  |
|  | WORiB(2) | wRi_009980-wRi_010580 | 72.115 |  |
|  | WORiC | wRi_006620-wRi_007230; | 67.515 |  |
|  |  | wRi_007550-wRi_007660 | 12.948 |  |
| *w*Tpre | WO-Island 1 | wTpre_69-76 | 3.163 | 28.317 |
|  | WO-Island 2 | wTpre_1393-1428 | 25.154 |  |

**Supplementary table 2**: Characteristics of *Wolbachia* strains used for comparisons.

| **Strain** | **Sg** | **Host** | **Effect on host** | **f/o** | **Reference** | **Accession nr.** |
| --- | --- | --- | --- | --- | --- | --- |
| *w*Au | A | *Drosophila simulans* | viral protection | f | Sutton et al. 2014 | GCA_000953315.1 |
| *w*Ha | A | *Drosophila simulans* | CI | f | Ellegaard et al. 2013 | GCA_000376605.1 |
| *w*Mel | A | *Drosophila melanogaster* | low CI/ antiviral | f | Wu et al. 2004 | GCA_000008025.1 |
| *w*Rec | A | *Drosophila recens* | CI/MK other host | f | Metcalf et al. 2014 | GCA_000742435.1 |
| *w*Ri | A | *Drosophila simulans* | CI | f | Klasson et al. 2009 | GCA_000022285.1 |
| *w*Uni | A | *Muscidifurax uniraptor* | PI | o | Newton et al. 2016 | GCA_001983635.1 |
| *w*Bol1 | B | *Hypolimnas bolina* | MK/CI res | f | Duplouy et al. 2013 | GFA_000333775.1 |
| *w*Lcla | B | *Leptopilina clavipes* | PI | o | this study | GCA_001855655.1 |
| *w*No | B | *Drosophila simulans* | CI | f | Ellegaard et al. 2013 | GCA_000376585.1 |
| *w*Pip | B | *Culex quinquefasciatus* | CI | f | Klasson et al. 2008 | GCA_000073005.1 |
| *w*Tpre | B | *Trichogramma pretiosum* | PI | o | Lindsey et al. 2016 | GCA_001439985.1 |
| *w*VulC | B | *Armadillidium vulgare* | F | f | Leclercq et al. 2016 | GFA_001027565.1 |
| *w*Oo | C | *Onchocerca ochengi* | normal development | o | Darby et al. 2012 | GCA_000306885.1 |
| *w*Ov | C | *Onchocerca volvulus* | normal development | o | Cotton et al. 2016 | GFA_000530755.1 |
| *w*Bm | D | *Brugia malayi* | normal development | o | Foster et al. 2005 | GCA_000008385.1 |
| *w*Fol | E | *Folsomia candida* | PI/egg hatching | o | this study | GCA_001931755.2 |
| *w*Cle | F | *Cimex lectularius* | vitamin B production | o | Nikoh et al. 2014 | GCA_000829315.1 |
| *w*Ppe | L | *Pratylenchus penetrans* | NA | f | Brown et al. 2016 | GCA_001752665.1 |

Abbreviations are used for certain host manipulations: CI for cytoplasmic incompatibility, PI for parthenogenesis induction, F for feminization and MK for male killing. Column label abbreviations: Sg stands for supergroup, f/o stands for facultative or obligate. If information was not available these characteristics were labelled NA.
