# Supplementary material for: Genome expansion of an obligate parthenogenesis-associated Wolbachia poses an exception to the symbiont reduction model
Source: BMC Genomics. 2019 Feb 6;20:106. doi: 10.1186/s12864-019-5492-9 (PMC6364476; doi:10.1186/s12864-019-5492-9)
Supplement: Supplementary file 3 — Abbreviations Fig. 2. List of abbreviations of annotations within arrows in Fig. 2. (DOCX 96 kb) [file 12864_2019_5492_MOESM3_ESM.docx]

Abbreviations annotations within arrows figure 2

| AAA | AAA family protein |
| --- | --- |
| ABCt | ABC transporter permease/ATP-binding protein |
| ado | L-allo-threonine aldolase |
| amt | adenosine monophosphate-protein transferase |
| ANK | ankyrin repeat containing protein |
| ANK+ | ankyrin repeat containing protein with additional domains |
| dhg | UDP-glucose 6-dehydrogenase |
| Dnar | putative DNA repair protein |
| end | putative endonuclease/ HNH endonuclease family protein |
| GpA | Phage terminase large subunit GpA |
| GpC | Putative phage minor capsid protein C |
| GpD | Putative phage head decoration protein D |
| GpE | Putative phage major capsid protein E |
| GpJ | Putative phage baseplate assembly protein J |
| GpU | Phage tail assembly protein GpU |
| GpV | Phage baseplate assembly protein V |
| GpW | Putative phage baseplate assembly protein GpW |
| GpX | Phage tail protein X |
| GpZ | Putative phage minor tail protein Z |
| Heli | Helicase SNF2 family |
| hsW | GpW-like phage protein |
| LCD | Phage late control gene D protein |
| ligA | DNA ligase (NAD(+)) LigA |
| met | Phage related DNA methylase |
| mfft | major facilitator family transporter |
| mtf | methyltransferase |
| PAAR | PAAR-family protein |
| pap | phage anti-repressor protein |
| Pat | patatin |
| phc | putative phage head-tail connector protein |
| phyH | Phytonoyl-CoA dioxygenase PhyH |
| pld | phospholipase D-like protein |
| plp | Putative phage portal protein, lambda family |
| pmp | putative membrane protein |
| ppp | putative phage related protein |
| ppr | putative phage repressor |
| ppt | putative phage tail protein |
| pseu | pseudogene |
| pss | putative type II secretion system protein |
| ptp | Putative phage tail protein |
| radC | DNA repair protein radC |
| res | putative resolvase |
| Res | resolvase |
| rhuM | putative virulence factor RhuM |
| rnap | putative RNA polymerase |
| rnh | ribonuclease H-like protein |
| rSAM | radical SAM protein |
| S-70 | sigma 70 |
| TAC | Phage tail assembly chaperone protein |
| tox | toxin |
| tra | transposase |
| trf | nucleotide-diphospho-sugar transferase |
| TSP | Phage major tail sheath protein |
| TTM | Phage tail tape measure protein |
| TTP | Phage tail tube protein |
| ucp | uncharacterized protein |
| uni | unique protein |
| vir | putative virulence protein |
| WDr | WD repeat domain protein |
